# Geographical and temporal distribution of cancer survival in teenagers and young adults in England

**DOI:** 10.1038/sj.bjc.6605410

**Published:** 2009-11-03

**Authors:** M Geraci, T O B Eden, R D Alston, A Moran, R S Arora, J M Birch

**Affiliations:** 1 Cancer Research UK Paediatric and Familial Cancer Research Group, The Medical School, University of Manchester, Stopford Building, Oxford Road, Manchester M13 9PL, UK; 2 Academic Unit of Paediatric and Adolescent Oncology, University of Manchester and Teenage Cancer Trust Young Oncology Unit, Christie Hospital NHS Foundation Trust, Withington, Manchester M20 4BX, UK; 3 North West Cancer Intelligence Service, Christie Hospital NHS Foundation Trust, Withington, Manchester M20 4QL, UK

**Keywords:** cancer survival, socioeconomic deprivation, spatial and temporal distribution, teenage and young adult cancer

## Abstract

**Background:**

Between 1979 and 2001, an analysis of cancer survival in young people in England, aged 13 to 24 years, showed overall improvements. However, for some diagnostic groups, little or no increases were observed. The aim of this study was to analyse the regional distribution of cancer survival in teenagers and young adults in England in order to identify patterns and potential for improvements at a regional scale.

**Methods:**

We examined geographical and temporal patterns in relative survival in cancer patients aged 13–24 years in England during the time period 1979–2001. Cancer cases were grouped according to an internationally recognised morphology-based diagnostic scheme.

**Results:**

For most diagnostic groups, there was little variation in survival between regions, except for testicular germ cell tumours (*P*=0.006) and colorectal carcinoma (*P*=0.002). For certain diagnostic groups, the temporal pattern in survival differed between regions. However, in regions that showed poor survival during the early part of the study period, greatest improvements were observed in groups such as acute lymphoid leukaemia, acute myeloid leukaemia, testicular tumours and melanoma.

**Conclusion:**

In conclusion, there was a reduction in the differences in survival between regions during the study period.

In the United Kingdom, cancer in teenagers and young adults (TYAs) aged 13–24 years is a major cause of both morbidity and mortality in this age range. In 2005, just under 1700 newly diagnosed cases in young people aged 15–24 years were registered in England alone (Office for National Statistics, 2008). Overall and for specific types of tumours, the geographical distribution of cancer incidence in TYAs was reported to show considerable differences between English regions, which may provide important clues for aetiology and potential for disease prevention (Alston *et al*, 2007). The assessment of regional variations in cancer incidence and survival is essential for efficient service planning.

Between 1979 and 2001, cancer survival in young people aged 13–24 years has improved over time in England (Birch *et al*, 2008). However, for some diagnostic groups, little or no improvements were seen. In addition, unlike cancers in older adults, most of the cancers predominant in this age range showed no association between survival and socioeconomic deprivation (Coleman *et al*, 1999; Quinn *et al*, 2005; Birch *et al*, 2008). In older adults, regional variations in survival have been reported for cancers diagnosed during 1971–1990, particularly in the earlier years (Coleman *et al*, 1999; Quinn *et al*, 2005). No comparable study has been carried out specifically in TYA cancer.

The aim of this study was to characterise the distribution of TYA cancer survival in England by Government Office Region (GOR) over time in order to provide baseline data at a regional scale and identify potential for improvements. Such a study is timely, given the impending implementation of national guidance on management of cancer in young people aimed at improving outcomes (National Collaboration Centre for Cancer, 2005).

## Methods

Patients diagnosed with any neoplasm, aged 13–24 years, in England during the period 1979–2001 (followed up to 31 December 2003) were included in this study. National cancer registration data on individual eligible cases were supplied by the National Cancer Intelligence Centre, Office for National Statistics, London. Data items provided included date of birth, diagnosis and follow-up, gender, histological type and primary site of cancer, GOR of residence, vital status and the Townsend deprivation index (TDI) score (Townsend *et al*, 1998) for census ward of residence of the patient at the time of diagnosis. Cases diagnosed from 1979 to 1995 were allocated 1991 census ward TDIs and those from 1996 to 2001 were allocated 2001 census ward TDIs. The relationship with TDI was analysed by grouping census wards into quintiles, such that the expected incidence for all cancers across England was the same in each quintile. The quintiles were ranked from 1 to 5, wherein 1 represented the most affluent and 5 the most deprived (Alston *et al*, 2007). The TDI score is thus standardised within each census.

Cases with vital status unknown, that is, patient record not traced at the National Health Service Central Register, were excluded from analysis, as were cases with a survival time of zero, that is, patients who were diagnosed and who died on the same day or postmortem diagnosis. These exclusion criteria were similar to those applied by Coleman *et al* (1999) in their publication. Cases lost to follow-up, for example, patients who emigrated, were included up to the date at which they were last known to be alive. The number of cases excluded for these reasons represented ∼4% of all eligible cases.

For cases registered from 1979 to 1994, cancer diagnoses were coded according to the International Classification of Diseases for Oncology, first edition (ICD-O1) (World Health Organization, 1976), and the ninth revision of the International Classification of Diseases (ICD 9) (World Health Organization, 1977). For cases registered between 1995 and 2001, diagnoses were coded according to ICD-O, second edition (ICD-O2) (Percy *et al*, 1990), and ICD tenth revision (ICD 10) (World Health Organization, 1992).

Cancer cases in 13–24-year olds were grouped according to the morphology-based diagnostic scheme described by Birch *et al* (2002). All diagnostic groups with <500 patients alive at least 1 day after diagnosis were excluded. The cancer groups included comprise acute lymphoid leukaemia (ALL), acute myeloid leukaemia (AML), non-Hodgkin's lymphoma (NHL), Hodgkin's lymphoma (HL), tumours of the central nervous system (CNS), osteosarcoma and Ewing tumour, soft tissue sarcomas (STSs), testicular germ cell tumours (GCTs), melanoma and carcinoma of ovary, cervix, colon and rectum.

Five-year relative survival in each diagnostic group was calculated by dividing observed survival by expected survival among comparable groups in the general population. The 5-year expected survival was derived from age-, sex-, year- and socioeconomic-specific national mortality rates for England (Coleman *et al*, 1999). Relative survival by GOR was examined using Poisson regression, as described by Dickman *et al* (2004). Four time periods (1979–84, 1985–89, 1990–95 and 1996–01) were defined, so that each one of them had an approximately equal number of incident cases. Geographical variations were modelled with random effects and the significance of variability was assessed using a likelihood ratio test statistic, after taking into account sex, age, time period and a quadratic follow-up temporal trend. Residual geographical variability after taking into account a trend in Townsend score quintiles was similarly assessed.

The significance level was set at 5%. Statistical analyses were performed using Stata v. 9.2 (StataCorp, 2005) and the software R (R Development Core Team, 2006).

## Results

Survival time was available for 33 274 (96%) out of 34 670 potential eligible patients. The total number of patients belonging to diagnostic groups of insufficient size, and thus excluded from the analysis, was 5204.

Between 1979 and 2001, statistically significant geographical variations in survival were seen for testicular GCTs (*P*=0.006) and colorectal carcinoma (*P*=0.002) (Table 1). Geographical variability was marginally significant for NHL (*P*=0.045) (Table 1). However, the residual geographical variability for the latter was not significant after considering a trend by socioeconomic deprivation (TDI). For all other groups, there was no significant evidence of differences in survival by GOR in the overall time period between 1979 and 2001.

**Figure 1:**
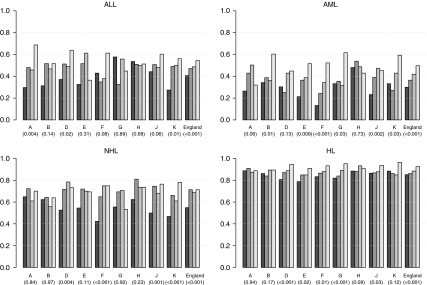
Five-year relative survival (%) of patients diagnosed with cancer at age 13–24 years in England, between 1979 and 2001, by region and time period. The *P*-value for time trend is reported in brackets. A, North East; B, North West; D, Yorkshire and Humber; E, East Midlands; F, West Midlands; G, East; H, London; J, South East; K, South West; ALL, acute lymphoid leukaemia; AML, acute myeloid leukaemia; NHL, non-Hodgkin's lymphoma; HL, Hodgkin's lymphoma. 
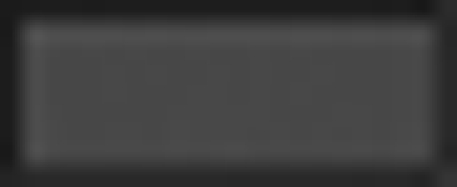
 1979–1984, 
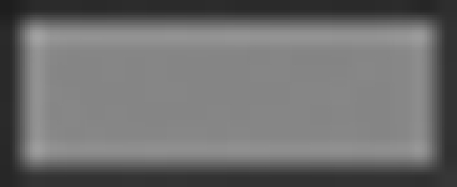
 1985–1989, 
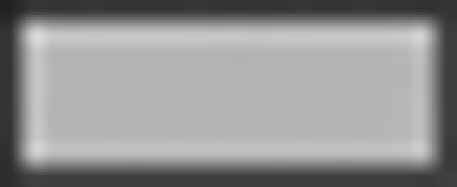
 1990–1995, 
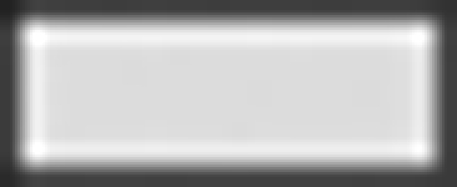
 1996–2001.

Between 1979 and 2001, 5-year relative survival for patients with ALL increased from 41% to 55% overall (*P*<0.001) (Figure 1). However, for individual regions, significant increases over time in survival occurred only in North East, Yorkshire and Humber and South West regions . Each of these regions had a lower survival for ALL than did England as a whole in the earliest time period but a higher survival in the latest time period. The highest increase was seen in North East, 30% in 1979–84 and 69% in 1996–2001 (the highest level among regions in this time period).

The relative survival of patients with AML across England increased from 30% in 1979–84 to 50% in 1996–2001 (*P*<0.001) (Figure 1). Significant upward temporal trends in AML survival were seen in North West, East Midlands, West Midlands, East, South East and South West regions. East and West Midlands were also the regions in which AML survival showed the steepest increase over time. These two regions showed the lowest survival in the earliest time period. In North West, East and South West regions, patients with AML had a survival of 60% in 1996–2001, the highest in this time period.

Overall, patients with NHL experienced a 55% survival in the time period between 1979 and 1984, increasing to 71% in 1996–2001 (*P*<0.001) (Figure 1). A significant increase in survival was seen in Yorkshire and Humber, West Midlands, South East and South West regions. For these GORs, the average rate of change in survival over time differed from that of the other GORs (*P*<0.001). In North East, North West, East Midlands and East regions, as well as in London, relative survival did not increase significantly. These regions, with the exception of London, had survival equal to or greater than the national average in 1979–84, but equal to or lower than the national average in 1996–2001. The average number of patients diagnosed with NHL in the first and last time period was, respectively, 38 and 55 per GOR.

The relative survival of patients with HL increased from 85% in 1979–84 to 93% in 1996–2001 (*P*<0.001) (Figure 1). However, the temporal pattern differed between regions. For Yorkshire and Humber and East regions, the rate of increase in survival over time was greater than that for the other regions (*P*<0.001). For North East, North West, London and South West regions, there was no evidence of increase in survival over time, but each of these regions showed equal or higher survival from HL than did England during the earliest period.

**Figure 2:**
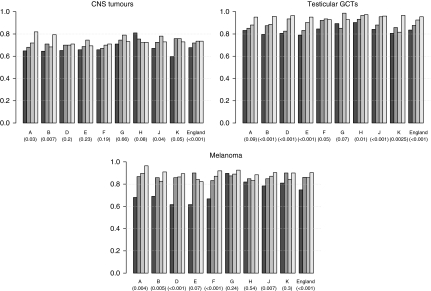
Five-year relative survival (%) of patients diagnosed with cancer at age 13–24 years in England, between 1979 and 2001, by region and time period. The *P*-value for time trend is reported in brackets. A, North East; B, North West; D, Yorkshire and Humber; E, East Midlands; F, West Midlands; G, East; H, London; J, South East; K, South West; CNS, central nervous system; GCTs, germ cell tumours. 
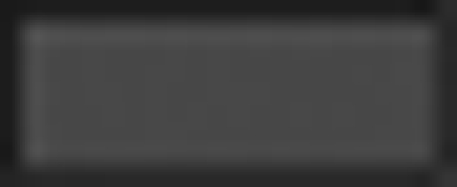
 1979–1984, 
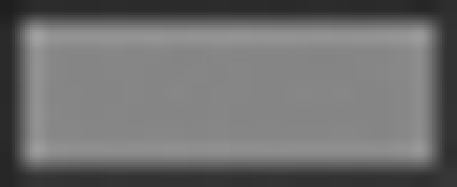
 1985–1989, 
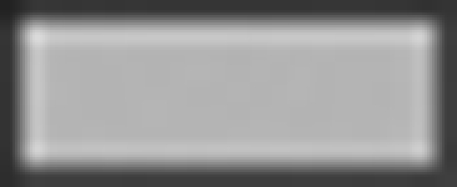
 1990–1995, 
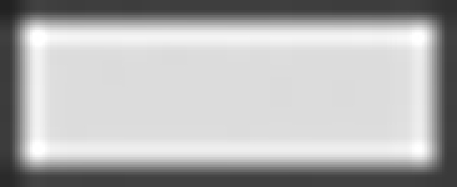
 1996–2001.

Five-year relative survival for CNS tumours in England was 68% in 1979–84, increasing to 72% in the following period and to 74% in 1996–2001 (*P*<0.001) (Figure 2). Survival in North East and North West regions increased from 65% in 1979–84 to around 80% in 1996–2001. These regions, together with South East and South West, showed on an average, a rate of increase in survival greater than that of the other GORs (*P*=.01). In contrast 5-year survival of CNS patients in London did not vary significantly over time (*P*=0.08).

Overall, relative survival for patients with testicular GCT increased from 84% to 96% between the first and last time period (*P*<0.001) (Figure 2). North West, Yorkshire and Humber, East Midlands, South East and South West regions had the most rapid increases over time in survival, but from lower starting points than many other regions.

Survival of patients diagnosed with melanoma between 1979 and 2001 in England increased from 75% in the first time period to 90% in the last time period (*P*<0.001) (Figure 2).

Regions of the North East, North West, Yorkshire and Humber and West Midlands, in which the 5-year survival rate from melanoma was below the national average in 1979–84, had the greatest increases over time (Figure 2). In contrast, survival in East Midlands, East, London and South West regions did not vary significantly between 1979–84 and 1996–2001. However, for the East, survival was consistently high throughout the four time periods.

**Figure 3:**
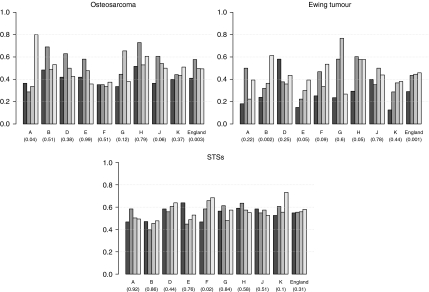
Five-year relative survival (%) of patients diagnosed with cancer at age 13–24 years in England, between 1979 and 2001, by region and time period. The *P*-value for time trend is reported in brackets. A, North East; B, North West; D, Yorkshire and Humber; E, East Midlands; F, West Midlands; G, East; H, London; J, South East; K, South West; STSs, soft tissue sarcomas. 
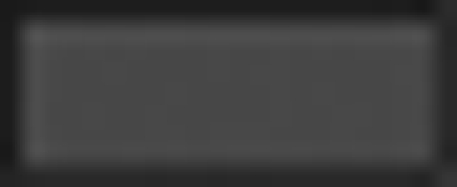
 1979–1984, 
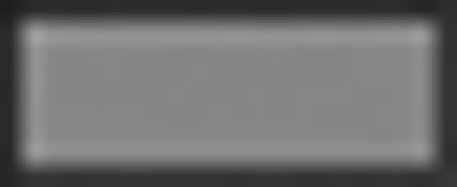
 1985–1989, 
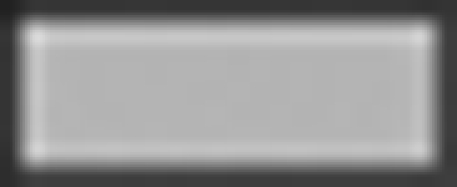
 1990–1995, 
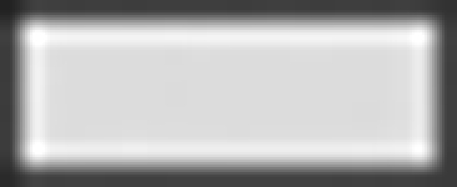
 1996–2001.

Patients diagnosed with osteosarcoma in England between 1979 and 1984 had a 5-year relative survival of 41%, which increased to 49% between 1996 and 2001 (*P*=0.003) (Figure 3). None of the GORs, with the exception of the North East, showed a significant variation in survival over time. The average number of incident cases diagnosed in the first and last period was, respectively, 36 and 28 per GOR.

Patients diagnosed with Ewing sarcoma in England between 1979 and 1984 had a 5-year relative survival equal to 29%, which increased to 46% between 1996 and 2001 (*P*=0.001) (Figure 3). Such an increase was mostly led by the North West region, with relative survivals equal to 24% in the first period and 65% in the last period.

Five-year relative survival for STS patients did not vary significantly over time between 1979 and 2001 (*P*=0.31) (Figure 3). However, West Midlands had a positive trend, increasing from 47% in the first time period to 69% in the last time period, at a rate significantly different from the rest of England (*P*=0.03).

**Figure 4:**
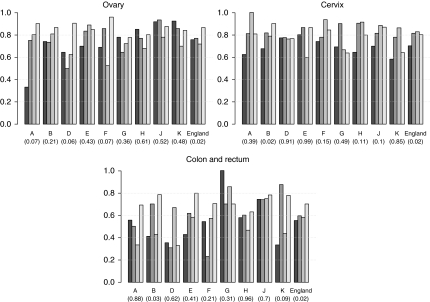
Five-year relative survival (%) of patients diagnosed with carcinoma at age 13–24 years in England, between 1979 and 2001, by region and time period. The *P*-value for time trend is reported in brackets. A, North East; B, North West; D, Yorkshire and Humber; E, East Midlands; F, West Midlands; G, East; H, London; J, South East; K, South West. 
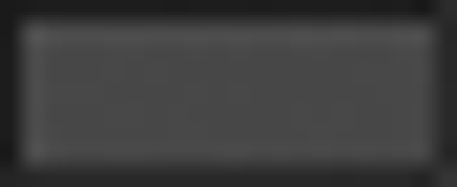
 1979–1984, 
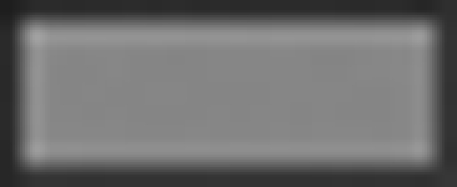
 1985–1989, 
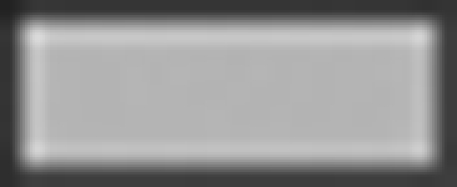
 1990–1995, 
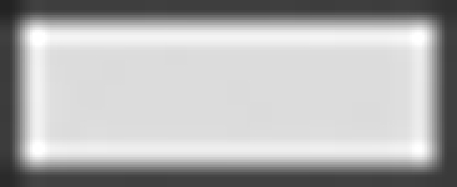
 1996–2001.

The relative survival of patients with carcinoma of the ovary, cervix and colorectum increased overall between 1979–84 and 1996–2001 (*P*=0.02) (Figure 4). However, at the regional level, a significant increase in survival over time only occurred in the North West region for cervical and colorectal carcinoma.

## Discussion

This study presents an analysis of the geographical and temporal distribution of relative survival from cancers that characterise TYAs in the age range of 13–24 years at the population level, using national data over a 23-year span. Factors including age, gender, time period and socioeconomic deprivation were taken into account. We found significant regional variations in relative survival for testicular GCTs and colorectal carcinoma. For several diagnostic groups under study, trends over time did not follow the same pattern regionally.

Quinn *et al* (2001) reported differences in survival by region for adult patients (15 years and over) diagnosed between 1986 and 1990. However, the TYA age group was not analysed separately and, furthermore, data were presented by ICD site and not by morphological diagnosis.

In a recent study based on present data, Birch *et al* (2008) analysed trends in survival by demographic groups and time periods at the national level. Overall, survival among TYAs with cancer has improved during the period 1979–2001 (Birch *et al*, 2008). This study also showed that for most cancers, there was no association between deprivation and survival but for leukaemias and carcinomas (mainly colorectal and head and neck carcinomas), there was a trend of poorer survival with increasing deprivation. However, in a separate study, we showed significant variations in the incidence of many TYA cancers by region and by deprivation (Alston *et al*, 2007). The question of possible variations in survival by geographical region therefore arises. Other previous studies of survival from cancer in TYAs deal with cases at the national level (Gatta *et al*, 2003; Steliarova-Foucher *et al*, 2004) or in several states in the United States combined (Bleyer *et al*, 2006). Furthermore, in those studies, different age ranges and/or time periods were considered. No previous studies have addressed survival patterns at the regional level within countries.

For many common adult onset cancers (breast, lung, colon and rectum), there exists an association between lifestyle factors such as tobacco smoking or poor diet and socioeconomic deprivation. The geographical variations in incidence of malignancies that are aetiologically linked to such factors are often the result of different levels of deprivation in different areas (Quinn *et al*, 2001). Marked differences in survival between 1971 and 1995, according to deprivation category, were observed for many of the major cancers at older ages, but such differences were not present for childhood cancers (Coleman *et al*, 1999).

In this study, the results show that for some diagnostic groups, variations in survival between regions cannot be entirely attributed to socioeconomic differentials, insofar as the latter are fully reflected by the Townsend score. Although lifestyle factors may have a role in this, other factors such as access to medical healthcare, referral patterns and clinical management should be considered and investigated (Birch *et al*, 2008).

Improvements in survival from ALL were seen in England overall, but the pattern by time period varied between regions, with significant increases seen in only three regions. The overall 5-year survival of 55% in England in the most recent time period is considerably less than the comparable figure of 83% for children (Stiller, 2007). Acute lymphoid leukaemia in adults has a worse prognosis than childhood ALL and there seems to be biological differences (Moorman *et al*, 2007). However, there is evidence that adolescents with ALL have a better outcome when treated on a paediatric protocol rather than an adult protocol (Ramanujachar *et al*, 2007). Some of the differences in survival trends between regions may be accounted for by referral patterns and proportions of TYA cases treated by paediatric and adult haematologists. The implementation of national guidance on management of TYAs with cancer should ensure a uniform approach to treatment with use of the most appropriate protocols.

Considerable improvements in survival over time concerned the group of testicular GCTs (Birch *et al*, 2008). Our results show that the 5-year relative survival in patients with testicular GCTs shows geographical variability after considering the deprivation index. However, rapid increases in survival between 1979 and 2001 have occurred, particularly in regions with poorer survival originally, so that in the most recent time period, all regions show survivals close to the national average.

Although for individual sites carcinomas are rare in TYAs, collectively, carcinomas form over 16% of all TYA cancers and form the second largest main group after lymphomas (Alston *et al*, 2007). Birch *et al* (2008) found a significant association between TDI and survival for some carcinomas, particularly colorectal and head and neck tumours. We observed substantial geographical differences in survival from colorectal carcinoma, which can be attributed only in part to a trend by socioeconomic deprivation. However, in the most recent time period, Figure 4 shows less regional variability in survival, although the numbers of cases in any single region and time period are small. This result raises the question as to what extent improvements in socioeconomic conditions and delivery of existing treatments affected survival during the study period rather than development and delivery of new treatments. Given the strong relationship between survival of patients with colorectal carcinoma and TDI score of residence (Birch *et al*, 2008), we can speculate that improvements in access to and delivery of existing treatment may have had a role in improving survival. However, because of the limitations of the currently available data, no specific analyses to address this question are possible. Nevertheless, new initiatives through the National Cancer Intelligence Network may make this possible in the future.

In conclusion, we analysed geographical patterns in cancer survival among TYAs on the basis of national data sets covering 23 years and more than 28 000 incident cases. Our results show that for most diagnostic groups, there is little variation in survival between regions. Analyses by time period show a general tendency for reduction in the differences between regions over time, with greatest improvements in those regions that showed poor survival during the early part of the study period. There has been a levelling up of survival rates across the country. The data also indicate that in those groups in which there has been little improvement in recent years (for example, bone and STSs), the problem is national and not because of poor performing regions. In cases in which regional variations are observed, the role of age-appropriate protocols and treatment in specialist units has to be established. Implementation of national guidance on management of young people with cancer in specified regional Principal Treatment Centres will, it is hoped, lead to improvements in outcome (National Collaboration Centre for Cancer, 2005). This study provides data on a regional basis with which treatment outcome in these developing regional specialist TYA cancer units and outcome of clinical trials can be compared. These outcomes will be of interest to specialists in adolescent oncology in other countries that are currently in the process of setting up specialist services for TYAs with cancer (Bleyer *et al*, 2007).

## Figures and Tables

**Table 1:** Five-year relative survival (%) and, in brackets, number of patients diagnosed with cancer at age 13–24 years in England between 1979 and 2001, by Government Office Region

	**North East**	**North West**	**Yorks and Humber**	**East Mids**	**West Mids**	**East**	**London**	**South East**	**South West**	**England**	***P*-value**	***P*-value after TDI**
ALL	47 (110)	44 (266)	48 (188)	46 (148)	43 (217)	47 (173)	52 (242)	50 (323)	46 (184)	47 (1851)	0.50	0.50
AML	38 (73)	42 (187)	36 (128)	37 (110)	31 (132)	40 (97)	48 (180)	39 (231)	41 (114)	40 (1252)	0.23	0.17
NHL	67 (121)	61 (310)	69 (239)	66 (169)	66 (266)	63 (217)	73 (399)	69 (415)	63 (245)	67 (2381)	0.045	0.06
HL	89 (282)	87 (782)	88 (594)	85 (457)	87 (643)	87 (655)	90 (873)	88 (1048)	89 (540)	88 (5874)	0.30	0.19
CNS	71 (261)	71 (694)	69 (547)	70 (428)	68 (461)	74 (421)	76 (651)	73 (845)	71 (483)	72 (4791)	0.08	0.08
Testicular GCTs	89 (175)	88 (503)	89 (397)	88 (337)	91 (391)	92 (386)	95 (519)	92 (662)	87 (395)	90 (3765)	0.006	0.009
Melanoma	87 (136)	84 (379)	84 (309)	82 (187)	84 (262)	90 (263)	84 (342)	86 (514)	86 (298)	85 (2690)	0.27	0.27
Osteosarcoma	46 (60)	54 (165)	51 (123)	45 (97)	36 (101)	44 (116)	60 (153)	49 (172)	44 (89)	49 (1076)	0.054	0.07
Ewing	31 (41)	41 (97)	44 (79)	27 (74)	42 (59)	45 (83)	54 (104)	43 (122)	30 (60)	41 (719)	0.06	0.07
STSs	52 (75)	44 (188)	60 (184)	52 (136)	60 (177)	56 (163)	59 (286)	56 (283)	60 (157)	56 (1649)	0.13	0.13
Ovarian carcinoma	73 (30)	80 (90)	72 (60)	82 (50)	76 (67)	73 (61)	78 (82)	87 (79)	84 (56)	79 (575)	0.50	0.43
Cervical carcinoma	81 (53)	79 (160)	77 (117)	78 (61)	84 (139)	70 (61)	81 (92)	81 (117)	73 (89)	79 (889)	0.50	0.50
Colorectal carcinoma	56 (32)	63 (80)	41 (58)	60 (45)	52 (55)	77 (36)	57 (71)	75 (127)	59 (54)	61 (558)	0.002	0.018

Abbreviations: ALL=acute lymphoid leukaemia; AML=acute myeloid leukaemia; CNS=central nervous system; GCTs=germ cell tumours; HL=Hodgkin lymphoma; Mids=Midlands; NHL=non-Hodgkin lymphoma; STSs=soft tissue sarcomas; TDI=Townsend deprivation index; Yorks=Yorkshire.
